# The readability of general practice websites: a cross-sectional analysis of all general practice websites in Scotland

**DOI:** 10.3399/BJGP.2020.0820

**Published:** 2021-04-07

**Authors:** Guy Rughani, Peter Hanlon, Neave Corcoran, Frances S Mair

**Affiliations:** Institute of Health and Wellbeing, University of Glasgow, Glasgow.; Institute of Health and Wellbeing, University of Glasgow, Glasgow.; Institute of Health and Wellbeing, University of Glasgow, Glasgow.; Institute of Health and Wellbeing, University of Glasgow, Glasgow.

**Keywords:** digital divide, general practice, health literacy, online systems, primary health care

## Abstract

**Background:**

General practice websites are an increasingly important point of interaction, but their readability is largely unexplored. One in four adults struggle with basic literacy, and there is a socioeconomic gradient. Readable content is a prerequisite to promoting health literacy.

**Aim:**

To assess general practice website readability by analysing text and design factors, and to assess whether practices adapted their website text to the likely literacy levels of their populations.

**Design and setting:**

Websites for all general practices across Scotland were analysed from March to December 2019, using a cross-sectional design.

**Method:**

Text was extracted from five webpages per website and eight text readability factors were measured, including the Flesch Reading Ease and the Flesch-Kincaid Grade Level. The relationship between readability and a practice population’s level of deprivation, measured using the Scottish Index of Multiple Deprivation (SIMD), was assessed. Overall, 10 design factors contributing to readability and accessibility were scored.

**Results:**

In total, 86.4% (*n* = 813/941) of Scottish practices had a website; 22.9% (*n* = 874/3823) of webpages were written at, or below, the government-recommended reading level for online content (9–14 years old), and the content of the remaining websites, 77.1% (*n* = 2949/3823), was suitable for a higher reading age. Of all webpages, 80.5% (*n* = 3077/3823) were above the recommended level for easy-to-understand ‘plain English’. There was no statistically significant association between webpage reading age and SIMD. Only 6.7% (*n* = 51/764) of websites achieved all design and accessibility recommendations.

**Conclusion:**

Changes to practice websites could improve readability and promote health literacy, but practices will need financial resources and ongoing technical support if this is to be achieved and maintained. Failure to provide readable and accessible websites may widen health inequalities; the topic will become increasingly important as online service use accelerates.

## INTRODUCTION

General practice websites are an increasingly important source of information and may provide the first point of interaction between patients and healthcare providers, yet, to the authors’ knowledge, there has been no large-scale research that assesses how understandable general practice websites are to their practice populations. Most practices in the UK have a website and there is impetus from the UK and Scottish government to increase the provision of services that GPs offer online, such as appointment booking and repeat prescription requests.^1^^,^^2^ In Scotland, it will soon be a requirement for all practices to make information and services available digitally;^2^ this process has been accelerated by the COVID-19 pandemic.^3^^,^^4^

The basis for general practice websites is commonly the practice leaflet, a contractually required document that provides information about the surgery’s services, opening times, appointments, prescriptions, data protection policy, and staff.^2^^,^^5^^,^^6^ A small number of website providers operate in the primary care market, with one company supplying nearly half of the general practice websites in the UK; consequently, many websites are similar in basic design and structure. As patients are sometimes required — and, increasingly, expect — to interact with health services via the internet, poorly produced websites can create a barrier to accessing health care.

The comprehensibility of text is often termed ‘readability’, which is defined as *‘the state or quality of being readable.’* 7 Text factors, such as word length or the number of syllables in a word, and design factors, such as line spacing and typeface, influence readability.^8^^–^10 Context is also important: familiar formatting — for example, having opening times written in table format — may help people understand complex information.^9^^,^^11^

Readability matters: in the latest major review of adult literacy 16.4%, or around 5.8 million people, in England and Northern Ireland score at the lowest level of proficiency in literacy (at or below Level 1) that is, they are only able to comprehend short sentences and identify single pieces of information if they were identical or synonymous with the information in a question;^12^ similar results were found in Scotland.^13^ Healthcare jargon and context adds complexity, even for those with otherwise good literacy levels.^14^ It has been found that 43% of written health information is too complex for UK adults to fully understand, a figure that rises to 61% when numerical information is included.^15^ Health literacy has been defined as the skills of individuals to *‘gain access to, understand, and use information to promote and maintain good health’*;^16^ however, in order to promote health literacy, text must be readable.^17^ Low basic literacy and low health literacy are associated with higher levels of socioeconomic deprivation.^13^^,^^18^ In Scotland, those living in the 15% of areas with the greatest levels of deprivation, according to the Scottish Index of Multiple Deprivation (SIMD), were twice as likely to only reach a basic level of literacy compared with those in all other areas.^13^ Computing literacy also varies with socioeconomic status: individuals in areas of greatest deprivation are least likely to be able to use, have access to, or know about online services.^19^^–^21

**Table:** How this fits in

GPs are encouraged to make more services available online, yet websites that are poorly written or produced can inadvertently create a barrier to accessing healthcare and widen health inequalities. In the largest study on website readability to date, all 813 general practice websites in Scotland were reviewed and most (77.1%) were more difficult to read than UK government-recommended limits. Websites were not adapted to their local population’s likely literacy levels, and only 6.7% met design and accessibility recommendations. Websites should be written in language suitable for people aged 9–14 years; simple measures can be taken to improve design and accessibility, but practices will need financial resources and technical support on an ongoing basis if this is to be achieved and maintained.

The NHS Information Standard states that, when creating information, providers should take *‘into consideration the health literacy and/or accessibility needs of the population it is aimed at.’* 22 Ensuring information is understandable is vital to enabling equitable access to health services.

This study explores general practice website readability by analysing text and design factors, and assessing whether readability varied according to the SIMD measure of a practice population’s level of deprivation.

## METHOD

### Data extraction

The authors used publicly available information from the Scottish Government — namely, the Information Services Division’s (ISD’s) list of all GP practices in Scotland, ranked by the percentage of each practice population’s level of multiple deprivation, as measured by the SIMD.^23^ The SIMD divides Scotland into 6976 neighbourhoods; each area is scored against 38 indicators of deprivation.^24^

Between January and July 2019, one author searched the internet to identify which of the 941 practices on the ISD/SIMD list had their own website. Directory-style entries on websites such as NHS Inform were not counted as independent general practice websites. Practice websites hosted by their local health board were included. If practices had merged with others and had a single group-practice website, the data were collected under the code of the practice whose physical site they shared; no data were collected for the relocated practice. Data were extracted from webpages that on discussion as a team were determined would be visited most often, such as the homepage (introductory page when clicking from a search result) and those with information on the following:

- appointments: how to make an appointment with a doctor;
- clinics and services: description of the clinics or extra services offered;
- repeat prescriptions: how to order repeat medicines; and
- new patient information: how to register.

### Text factors

The primary measures of text readability were the Flesch-Kincaid Grade Level (FKGL) and Flesch Reading Ease (FRE) scores.^25^ These are well-established tools, and proxies for gold-standard comprehension tests.^18^ They consider average sentence length and syllables per word,^19^ and both are widely used and freely available in word-processing software.^19^ Both formulae have correlation coefficients of >0.9 with comprehension tests.^20^

UK government website designers and literacy campaigners suggest that websites should be comprehensible by a 9–14 year old.^8^^,^^25^ Text should follow the principles of ‘plain English’ and:

- use short, everyday words;
- avoid jargon;
- be written in the first person; and
- use an active, rather than a passive, voice.^22^^,^^23^

The readability statistic target for ‘plain English’ is an FRE of ≥60/100.^22^

Six secondary measures were recorded, in line with recommendations from a previous review of readability:

- character count;
- characters per word;
- word count;
- words per sentence;
- sentences per paragraph; and
- paragraph count.^1^

One author checked each of the five webpages for all of the general practice websites. If practices did not have a separate webpage for the different areas of information (for example, appointments), but had the relevant text on another part of the website, that text was extracted. Where possible, only the main area of webpage text (‘body text’) was extracted; navigation information, headers, and footers were not assessed.

Body text was manually selected, copied, and pasted into Microsoft Word (2016) and formatting elements such as bulleted or numbered lists, headings, titles, tables, figures, and paragraph breaks were discarded.

Where there had been a bulleted or numbered list, full stops were added to the end of each line; without full stops, the software calculated the whole list as a single sentence, which was misleading as the purpose of a list was, generally, to improve readability.

A different researcher checked the readability statistics for 10% of the websites, selected at random. This revealed 100% agreement, so the first researcher extracted the remaining data. With R software (version 3.6.2), linear regression was used to model the SIMD ranking for each practice against the FKGL score.^26^

A significant change in readability score was considered to be one grade level — that is, one UK school year.

### Design factors

Design factors that contributed to readability and accessibility were also assessed. Informed by NHS England’s Information Standard, the UK government website’s design system, and recommendations from the Plain English Campaign, a 10 factor design score of desirable features was created (Box 1).^10^^,^^11^^,^^22^ Typeface size was not investigated because it adjusts automatically based on individual settings, making it difficult to reliably record. The appointments page (or equivalent section) was assessed, as it was thought that there would be appointments content on most websites and it has relevance to both new and existing patients. The body text (rather than the navigation information, header, or footer) was scored. A score out of eight was given if there were no images; a score out of 10 was given if images had been used. To allow for a true comparison of webpage scores, a scaled design score was calculated — each score was divided by the maximum possible score for that webpage, giving a final score of 0.0–1.0.

**Box 1.** Design score

**Basic factors (1 point each)** Use of sans-serif typeface in headings, main text, and captions Use of a single typeface in headings, main text, and captions ‘Scannable’ text — use of features such as subheadings, bullet lists, or paragraph breaks to divide information Bold: used for emphasis only No block capitals No italicised text Clear contrast between text and background colours Optimised for smartphone browsers — the webpage must automatically detect it is being viewed on a smartphone screen and adjust to the screen ratio so that it is usable **Additional items if images present (1 point each)** Captioned illustrations — all images should be captioned Alt text (a meaningful description of the image that screen-reading software can read aloud to aid users who are partially sighted) on illustrations

The first researcher scored each website’s appointments page or section, and the second scored a random 10% of pages. Discrepancies were discussed and a third researcher reviewed those that could not be resolved.

Whether design scores varied within, and between, website providers was assessed. R software (version 3.6.2) was used to calculate the scaled design scores, mean scaled score, and standard deviation for each provider, and the number of webpages that scored full marks.^27^ Using linear regression, the authors also assessed whether there was a correlation between the design score and the FRE readability statistic.

### Sensitivity analyses

Sensitivity analyses were conducted for both the readability and scaled design-score investigations by excluding webpages with <150 words.

## RESULTS

Table 1 gives details of the websites and anonymised providers. Of all 941 practices, 813 had a functioning website, 122 did not have a website, and six practices had merged with other practices (their web address re-directed to the new joint practice website).

**Table 1.** Practice websites and website providers

	***n***	**All practices on ISD list,** ***n*= 941, %**	**Unique practice websites,** ***n*= 813, %**
Practices on ISD 2016 list	941	—	—

Practices that had merged their websites with those of other practices	6	0.6	—

Practices with no website	122	13.0	—

Total number of unique practice websites	813	86.4	—

Practices with an appointment page/section to allow calculation of the design score	764	81.2	94.0

Website provider (anonymised and listed individually if ≥10 websites provided)			
A	33	3.5	4.1
B	65	6.9	8.0
C	19	2.0	2.3
D	417	44.3	51.3
E	35	3.7	4.3
F	32	3.4	3.9
G	13	1.4	1.6
H	11	1.2	1.4
I	50	5.3	6.2

Designed in house by the practice	80	8.5	9.8

Miscellaneous (designed by a web company providing<10 websites)	58	6.2	7.1

ISD = Information Services Division.

### Reliability

A random 10% of the ISD list of 941 practices (95 websites) were independently scored. There were no discrepancies in readability scoring. Of the 95 webpage design scores, seven had discrepancies of a maximum of 1 point (Cohen’s k coefficient: 0.98); these discrepancies were resolved by discussion.

### Readability statistics

**Figure 1.**
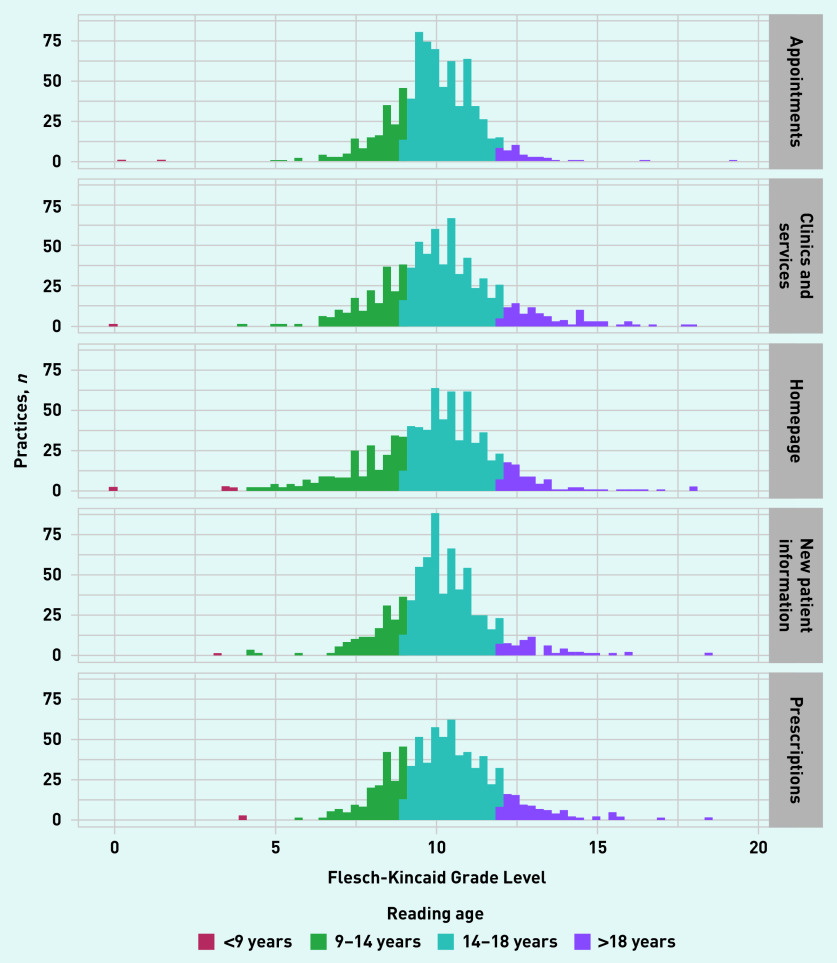
***Flesch-Kincaid Grade Level by webpage.*** ***Grade level has been converted to reading-age range.***

If all 813 functioning websites had five webpages with extractable data, there would have been 4065 potential webpages to analyse. Readability statistics were calculable for 94.0% (*n* = 3823/4065) of possible webpages (see Supplementary Table S1). Of all 3823 webpages, 77.1% (*n* = 2949) featured information that exceeded the recommended 9–14-year-old reading age for online content; 22.9% (*n* = 874/3823) featured information at, or below, the recommended age range. Figure 1 presents the FKGL scores (converted from the score’s grade level of education used in its native US to age equivalents) from all the practice websites, plotted by webpage type.

**Figure 2.**
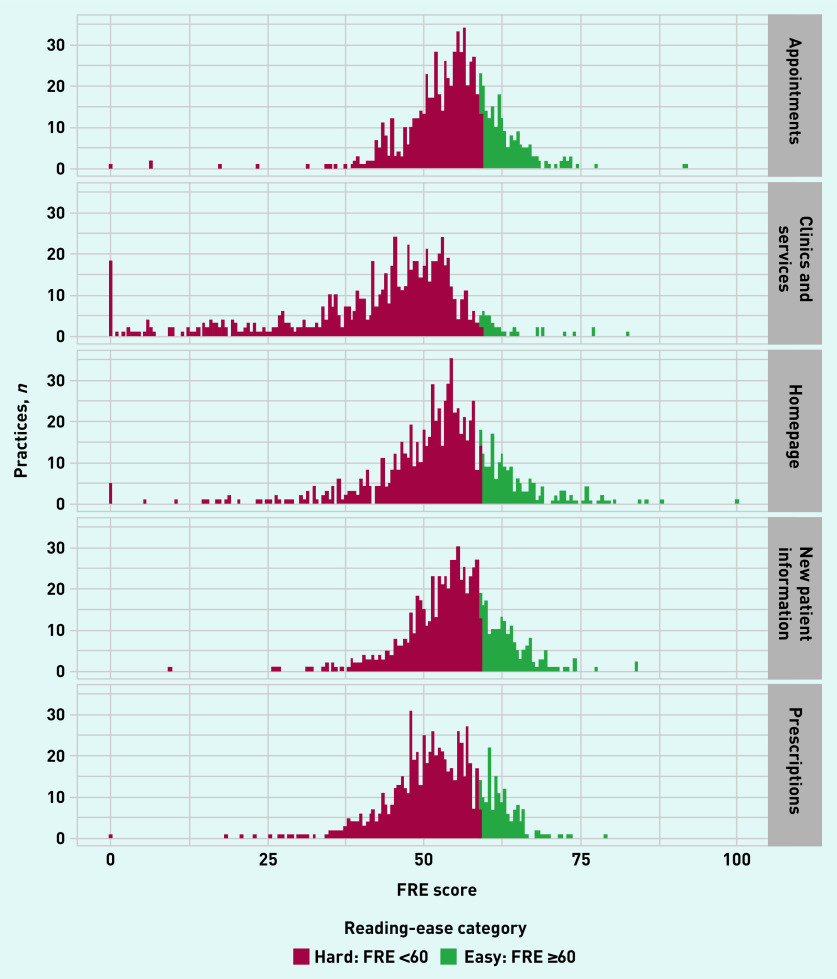
***Flesch Reading Ease score by webpage. Outlying peaks at ‘FRE = 0’ in ‘Clinics and services’ and ‘Homepage’ were removed when pages with <150 words were excluded, but the overall proportions above the FRE threshold remained consistent (see sensitivity analysis and Supplementary Figure S3). FRE = Flesch Reading Ease.***

The FRE results were similar (Figure 2). In total, 80.5% (*n* = 3077/3823) of webpages scored below the recommended FRE cut-off of ≥60 for ‘plain English’ (data not shown).

There was no statistically significant association between the webpage reading ages (FKGL converted to UK age ranges) and the practice population’s level of multiple deprivation (SIMD quintile) (data not shown); Supplementary Figure S1 presents the reading-age level for the five webpage types by SIMD quintile. Secondary readability statistics are reported in Supplementary Table S2.

### Design and combined scores

In total, 94.0% (*n* = 764/813) of practice websites had an appointments section (Table 1), allowing a scaled design score to be calculated. Of these, 6.7% (*n* = 51/764) scored full marks for design and accessibility (data not shown).

**Figure 3.**
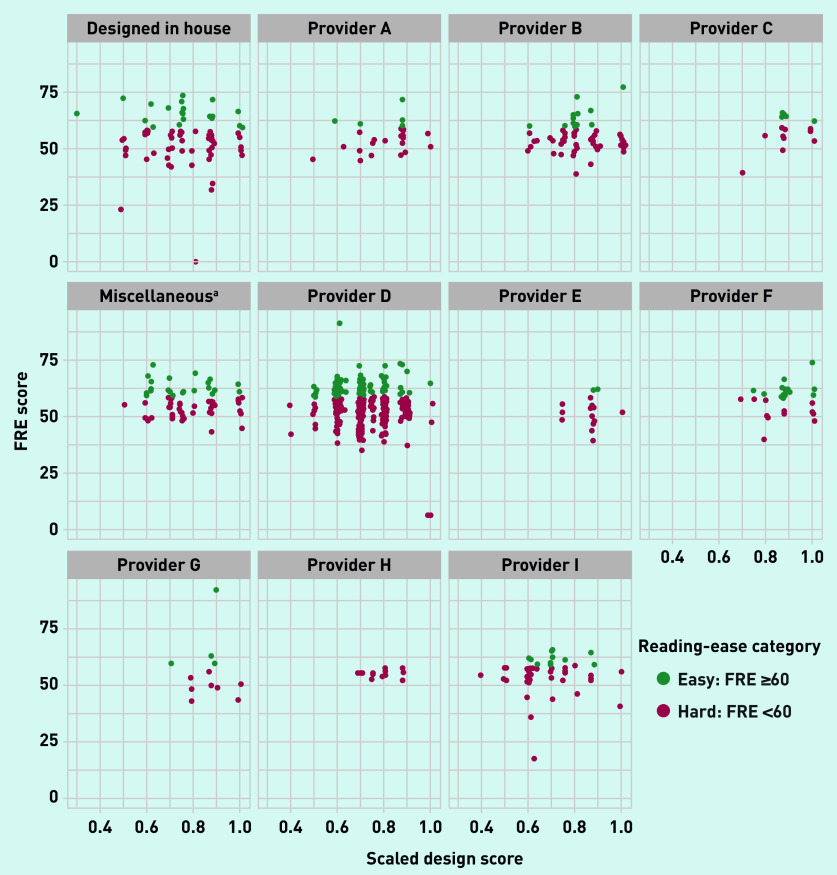
***Combined readability (Flesch Reading Ease) and scaled design scores by website provider/provider type.*** *^a^****Designed by a web company providing <10 websites. FRE = Flesch Reading Ease.***

There was a spread of scaled design scores for each provider (Figure 3), but a similar variation in mean scaled design scores between website providers (see Supplementary Table S3). Figure 3 presents the scaled design score for each appointment webpage by that webpage’s readability (classified as ‘hard’ or ‘easy’ to read). There was no statistically significant association between the design score and readability (FRE statistic), with a correlation of 0.004 and *P*-value from linear regression of 0.93 (data not shown).

### Sensitivity analysis

Webpages with <150 words were excluded and the analyses were repeated; results are shown in Supplementary Figures S2–S5. This removed the most extreme outlying readability scores (for example, ‘clinics and services’ that are visible in Figure 2), but the proportions of pages above the thresholds remained consistent (see Supplementary Figure S3). A slightly stronger correlation (0.04) was noted between the design score and FRE, but remained not statistically significant (*P* = 0.23). Supplementary Table S3 and Supplementary Table S4 detail further descriptions of the remaining outliers.

## DISCUSSION

### Summary

In Scotland, 86.4% of GP practices have a website; however, 77.1% (*n* = 2949/3823) of webpages include content that exceeds the recommended target reading age of 9–14 years old,^8^^,^^27^ and 80.5% (*n* = 3077/3823) include content that exceeds the target for ‘plain English’.^26^ There was no evidence that practice websites were adapted to meet the likely literacy levels of the populations they serve. Only 6.7% of websites met accessibility and design recommendations^28^ (Box 1). All website providers had evidence of suboptimal design in terms of readability and the market was dominated by a single provider using a limited number of website design templates; however, the spread in design scores across providers may demonstrate that practices retain some control over the readability of their output. Surprisingly, there was no association between text readability and the design scores, highlighting that a clear-looking website is not necessarily readable.

### Strengths and limitations

To the authors’ knowledge, this is the most comprehensive assessment of general practice websites to date, and the first to analyse design factors. Variability in website production required judgement to decide which text should be analysed, but there was minimal variation in scoring between researchers.

The main limitation was the use of readability scoring tools. The FRE and FKGL measures were designed for an US context and, although they are reliable, other measures are arguably better adapted to health care;^1^^,^^29^^,^^30^ the Flesch formulae, however, are the only ones embedded in commonly available software and were, therefore, the only practical option for this study.

Readability formulae can be misled by low word counts and special characters^1^ — for example, telephone numbers score as single difficult words.^1^^,^^31^^,^^32^ As recommended, the authors were consistent in the formatting that was permitted, and performed sensitivity analyses.^1^ Formulae also ignore word meaning and the added complexity of numerical information, so are a proxy for comprehensibility.^15^^,^^18^^,^^31^ The high volume of health-related language on general practice websites may mean the formulae underestimate the impact of poor health literacy — as an example, ‘gastric’ and ‘tummy’ are two-syllable words that score equally, yet may be differently understood.^1^

The ease of website navigation can be a barrier and it was not possible to establish a robust method of assessment. It is possible that seemingly readable websites may be difficult to use. User testing would clarify the link between proxy scores and the real-world usability and comprehensibility of websites but that process was beyond the scope of this study. The authors also did not have the capacity to investigate accessibility for people who do not speak or understand the English language.

### Comparison with existing literature

General practice website readability has been under-researched. One small study assessed the readability of 10 English practice websites; it reported that ‘most’ websites had an FRE score of 50–60, suggesting that half of the UK adult population would struggle to fully understand the content.^33^

Patient information leaflets (PILs) and online condition-specific information has been more widely studied; poor readability has been universally reported.^34^^–^38 In comparison with Protheroe *et al* ’s^17^ UK study of PILs in general practices, which used the same readability formulae, the study presented here found that a greater proportion of webpages featured content that exceeded the reading level of a 14 year old (77.1% for webpages versus 37.4% for PILs). Both studies found a similar proportion fell within the respective readability targets (23.0% for webpages versus 24.3% for PILs).^17^

### Implications for practice

The data presented here suggest most general practice websites across Scotland do not meet the standards recommended by the NHS, government, and literacy campaigners; in addition, there is no consistent evidence that practices in more socioeconomically deprived areas adjust the readability of their websites to meet the likely lower literacy levels of the populations that they serve, and vice versa.

It is possible that the hastened uptake of digital health due to the COVID-19 pandemic could exacerbate health inequalities, especially if literacy is not considered.^39^

In Scotland, a national template for practices to adapt is being considered. Although website design may improve, practices will need support to create accessible content; pre-population with user-tested accessible text could help, as could the development of an NHS style guide, like that developed by Government Digital Service.^25^

While awaiting national efforts, practices can take steps to improve the situation: the authors’ simple 10-point design score (Box 1) could be used as a guide. Flesch readability scores are freely available, but readability can be improved by asking, while editing the website or creating the content, ‘could a 9-year-old child understand this?’. Removing medical terms only understood by those in the medical profession is critical.^9^

The authors’ assumption is that readability and design improvements promote comprehension and health literacy, but this can only be assessed by testing websites with their target users.^14^^,^^31^^,^^40^

Practices will require financial resources and technical support on an ongoing basis to enact and maintain such recommendations, but failure to do so may inadvertently widen health inequalities.

In a time of scarce resources, partnerships between patient participation groups, literacy charities/campaign bodies, government, and practices may be necessary to ensure digital changes are inclusive.
